# 210-Day Kinetics of Total, IgG, and Neutralizing Spike Antibodies across a Course of 3 Doses of BNT162b2 mRNA Vaccine

**DOI:** 10.3390/vaccines10101703

**Published:** 2022-10-12

**Authors:** Chin Shern Lau, May Lin Helen Oh, Soon Kieng Phua, Ya-Li Liang, Tar Choon Aw

**Affiliations:** 1 Department of Laboratory Medicine, Changi General Hospital, 2 Simei Street 3, Singapore 529889, Singapore; 2 Department of Infectious Diseases, Changi General Hospital, Singapore 529889, Singapore; 3 Department of Medicine, National University of Singapore, Singapore 117599, Singapore; 4 Academic Pathology Program, Duke-NUS Graduate Medical School, Singapore 169857, Singapore

**Keywords:** SARS-CoV-2, booster vaccination, kinetics

## Abstract

Introduction: We tested the total spike antibody (S-Ab), IgG/IgM S-Ab, and neutralizing antibody (N-Ab) responses of COVID-19-naïve subjects from before their first BNT162b2 vaccination up to 210 days after boosting. Methods: We studied 136 COVID-19-naïve subjects who received three doses of the Pfizer mRNA vaccine (39 males, 97 females, mean age 43.8 ± 13.5 years) from January 2021 to May 2022. Serum was assessed for total S-Ab (Roche), IgG/M (Abbott), and N-Ab (Snibe). Results: Peak antibody levels were measured 20-30 days after each dose, with booster dosing eliciting significantly higher peak antibodies than the second dose: total S-Ab 2219 vs. 19,551 BAU/mL (difference 16,667 BAU/mL, *p* < 0.0001); IgG 2270 vs. 2932 BAU/mL (difference 660 BAU/mL, *p* = 0.04); and N-Ab 3.52 vs. 26.4 µg/mL (difference 21.4 µg/mL, *p* < 0.0001). Only IgM showed a lower peak post-booster antibody titer (COI 2.11 vs. 0.23, difference 1.63, 95% CI 1.05 to 2.38, *p* < 0.0001). By 180–210 days after the second or third vaccination, total S-Ab/IgG/N-Ab had decreased by 68.7/93.8/73.6% vs. 82.8/86.3/79.5%. The half-lives of IgG and N-Ab antibodies were longer after the third vaccination (IgG: 65 vs. 34 days, N-Ab: 99 vs. 78 days). Conclusion: Total S-Ab/IgG/N-Ab showed a greater increase post-booster, with IgG/N-Ab having a longer half-life.

## 1. Introduction

Although it has been nearly three years since the start of the COVID-19 pandemic, many countries around the world are still struggling to vaccinate their populations [1]. Vaccination is essential to control the spread of SARS-CoV-2 and has clear benefits in preventing COVID-19 related morbidity and mortality. Vaccinations were estimated to have prevented 14.4 million deaths during the first year of the pandemic [2] and to have reduced infection and hospitalization rates across several populations [3]. Even in adolescents aged 12–17 years, a single dose of Pfizer BNT162b2 mRNA vaccine reduced infection risk by 63.7% after 61–90 days [4].

However, a troubling concern is that even after two inoculations, vaccine-induced antibody responses wane over time. One study showed a drop to 25% residual IgG spike antibody (S-Ab) reactivity after 82.6–89.4 days, regardless of the initial IgG levels [5]. Even total S-Ab levels decreased by 42.7% 79 days after a second dose of the BNT162b2 mRNA COVID-19 vaccine [6]. This is concerning, as reduced antibody levels indicate a reduction in anti-viral protection, with COVID-19-naïve BNT162b2 vaccinees experiencing a decrease in vaccine effectiveness from 85 to 51% 201 days after their second dose regardless of the vaccination interval [7]. With the advent of several variants of concern, this can result in an increase in breakthrough infections even in vaccinated individuals [8].

To counter this, several countries have encouraged the use of a third, “booster” vaccination. Indeed, the CDC [9] now recommends three doses of Pfizer vaccine not just in adults but in children and adolescents as well. The reported performance of booster vaccination programs is quite good, even in the current climate where the Omicron variant is predominant. In a study of the real-world effectiveness of booster vaccination in the US [10], during a period of Delta predominance, vaccine efficacy (against confirmed COVID-19 infection) was 76% ≥180 days after dose two but rose to 94% ≥14 days after a booster dose of Pfizer vaccine. The booster vaccine also claimed an effectiveness of 82% during a period of Omicron variant predominance, with an efficacy against hospitalization of 90%. Even heterologous booster regimens demonstrated impressive results: in a large Chilean study [11], in patients who had received an initial two doses of CoronaVac, a booster dose of BNT162b2 vaccine generated an estimated vaccine efficacy of 96.5% with an adjusted vaccine effectiveness of 96.1% against hospitalization 2 weeks after a third dose. However, few studies have reported the extended antibody kinetics after a third dose of vaccine. This would have a bearing on protective public health measures. 

In our country, healthcare workers were encouraged to take a third booster vaccination between October and November 2021. We previously reported the early antibody responses in healthcare workers after their third vaccination [12]. We now report on their progress after booster vaccinations.

## 2. Methods

### 2.1. Study Participants

We studied 136 subjects who received 3 doses of the Pfizer mRNA vaccine (39 males, 97 females, mean age 43.8 ± 13.5 years) from January 2021 to May 2022. During this period, our country experienced two waves of SARS-CoV-2 variants: Delta from August to November 2021, and Omicron from December 2021 onwards [13]. All subjects were COVID-19 naïve, with no reported COVID-19 infections during the entire study period, which was evidenced by negative SARS-CoV-2 nucleocapsid antibodies (Roche total anti-SARS-CoV-2 nucleocapsid antibody assay) at the beginning of the study and at all time points tested to account for asymptomatic/pauci-symptomatic infections. Total S-Ab (Roche), IgG S-Ab (Abbott), and neutralizing antibodies (N-Ab) (Snibe) levels were tested at set time points after vaccination up to 210 days post-booster. IgM S-Ab (Abbott) levels were tested up to 60 days post-booster. Due to differences in subject vaccination schedules, the number of samples differed at each time point. All samples obtained in this study were de-identified and anonymized.

### 2.2. Methods and Materials

Serum at each time point was obtained and stored at −70 degrees Celsius if not immediately analyzed. Frozen samples were thawed for 1 h at room temperature and vortexed prior to analysis. The Roche Elecsys Anti-SARS-CoV-2 S assay (a quantitative double-antigen sandwich electro-chemiluminescent immunoassay performed on the Roche Elecsys e801 auto-analyzer) and the Snibe competitive quantitative N-Ab assay (performed on the Snibe Maglumi) have been previously described in prior studies from our laboratory [12]. We also utilized the Abbott quantitative IgG and IgM SARS-CoV-2 S-Ab assays, both of which have been previously described [14,15]. The Roche total S-Ab assay reports titers in U/mL and is converted to WHO international units (BAU/mL = 0.97 × U/mL). Similarly, Abbott IgG is reported in AU/mL and is converted to WHO units (BAU/mL = 0.142 × AU/mL).

### 2.3. Statistical Analysis

Data were presented as medians with ranges where appropriate. No indeterminate or missing results were used. We utilized the Mann–Whitney U test to compare the antibody titers between different vaccination groups at each time point, with *p* < 0.05 considered statistically significant. Statistical analyses were performed with MedCalc Statistical Software (version 20.008, MedCalc Software Ltd., Ostend, Belgium). This work was part of a seroprevalence survey using deidentified, anonymized samples/data, and was thus exempt from our hospital’s Institutional Review Board. However, informed consent was obtained from all subjects involved, as they needed to provide blood samples at several time points. Compliance with STARD guidelines is enclosed (see Supplementary Table S1).

## 3. Results

### 3.1. Antibody Responses

**Figure 1:**
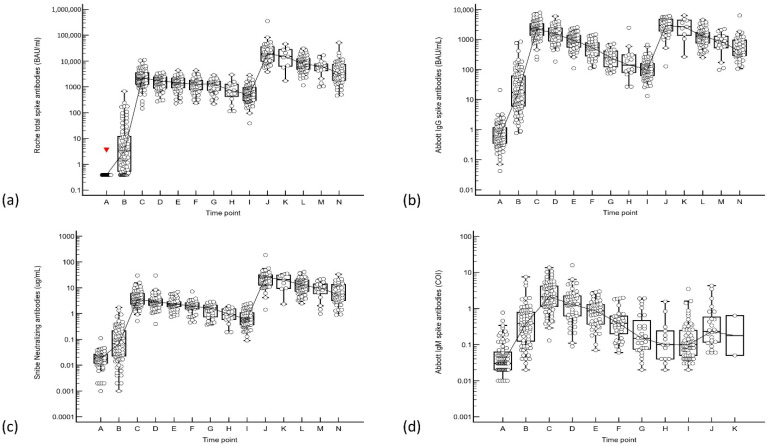
(**a**) Roche total spike antibody, (**b**) Abbott IgG, (**c**) Snibe neutralizing antibody and (**d**) Abbott IgM responses to 3 doses of Pfizer mRNA vaccine. Time points are represented by A: Baseline, B: 10 days post-dose 1, C: 20 days post-dose 2, D: 40 days post-dose 2, E: 60 days post-dose 2, F: 90 days post-dose 2, G: 120–150 days post-dose 2, H: 180–210 days post-dose 2, I: 240 days post-dose 2 to pre-dose 3, J: 20–30 days post-dose 3, K: 60 days post-dose 3, L: 90 days post-dose 3, M: 120–150 days post-dose 3, N: 180–210 days post-dose 3. Antibody levels are expressed on a semi-logarithmic scale.

Total S-Ab, IgG, and N-Ab increased appropriately after the second and third doses of mRNA/inactivated virus vaccine (see Figure 1 and Table 1). The median peak antibody levels (20–30 days post-vaccination) following the second and third dose of Pfizer vaccine were: total S-Ab levels of 2219 and 19,551 BAU/mL; IgG 2270 and 2932 BAU/mL; and N-Ab 3.52 and 26.4 µg/mL, respectively. 

Comparing the peak responses between the second and third vaccinations, the post-third dose peak was significantly higher for total S-Ab (difference 16,667 BAU/mL, 95% CI from 12,927 to 19,688, *p* < 0.0001); IgG (difference 660 BAU/mL, 95% CI from 29.9 to 1302, *p* = 0.0411); and N-Ab (difference 21.4 µg/mL, 95% CI from 15.0 to 24.6, *p* < 0.0001). The ratio of total:IgG S-Ab increased between the second and third vaccinations (from 0.98 to 6.67). However, the median peak IgM S-Ab levels 20–30 days post-booster were significantly lower than the second dose (COI 2.11 vs. 0.23, difference 1.63, 95% CI from 1.05 to 2.38, *p* < 0.0001).

### 3.2. Antibody Kinetics

We followed the antibody levels of total S-Ab, IgG, and N-Ab up to 210 days post-booster, and by 90 days after the second vaccination, all antibodies declined: IgG by 76.1%, N-Ab by 44.6%, and total S-Ab by 43.3%, respectively. By 180–210 days (6–7 months) after the second vaccination, the magnitude of antibody decline from peak levels was: IgG 93.8%, total S-Ab 68.7%, and N-Ab 73.6%, respectively. In contrast, the decline from peak antibody levels was lower after the third vaccination for IgG and N-Ab: 90 days after the third vaccination, IgG by 56.5% and N-Ab by 52.7%, 180–210 days, IgG by 86.3% and N-Ab by 79.5%. Even after 180–210 days of post-booster vaccination, all antibody levels were significantly higher than pre-booster (dose two day 240 to pre-dose three) titers (Total S-Ab 2750 BAU/mL, *p* < 0.0001; IgG 305 BAU/mL, *p* < 0.0001; N-Ab 4.75 µg/mL, *p* < 0.0001).

**Figure 2:**
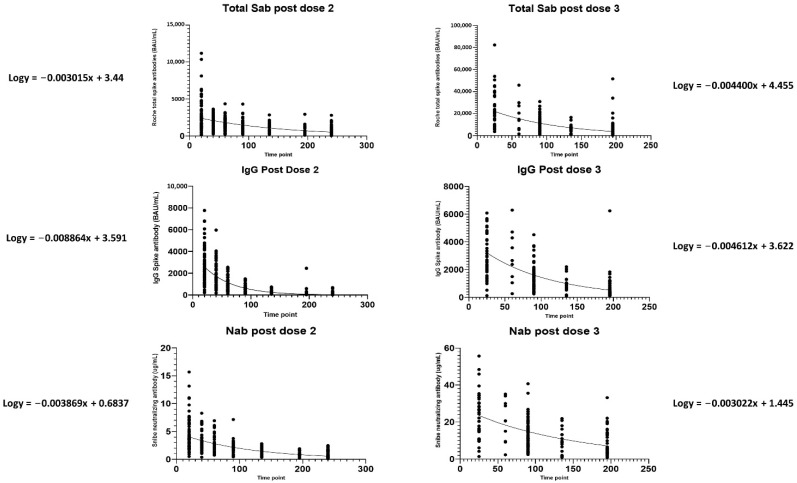
Non–linear regression analysis of the waning of total, IgG, and neutralizing antibodies after the second and third vaccinations, with associated regression equations.

We generated non-linear regression plots of the decline of total S-Ab/IgG/N-ab after the second vs. third inoculations. The half-lives (time to 50% antibody titer, AT50) of IgG and N-Ab antibodies were longer after the third vaccination (IgG: 65 vs. 34 days, N-Ab: 99 vs. 78 days). Interestingly, the total S-Ab displayed a shorter half-life after the third dose (69 days) compared to the second dose (100 days) (see Figure 2).

## 4. Discussion

Following the second and third doses of the vaccine, IgG, total S-Ab, and N-Ab all declined with time. This is supported by another study [16], where in 91 COVID-19-naïve subjects, total Roche S-Ab titers peaked 1 month after the third dose, but by 4 months (120 days), they had decreased by 79.3%. This trend is similar to our population (67.5% decrease by 120–150 days after booster). In another study of spike IgG responses in 94 vaccinees receiving three doses of Pfizer vaccine, D28/D84 IgG levels experienced a 52.3% decline [17]. The post-booster IgG levels in our study also had a similar decrease (56.5%) by 90 days. In spite of this, the antibody titers at 180–210 days post the third dose were still significantly higher than levels pre-booster. In our population, all antibodies (except IgM) experienced a significantly higher peak after the booster dose compared to the second dose. This was also demonstrated in 48 subjects of another study [5], with the majority of boosted patients showing a higher peak reactivity compared to the second dose (e.g., six individuals with an initial baseline IgG <500 BAU/mL after the second dose all showed higher reactivity, with half having IgG >2000 BAU/mL). Furthermore, we found that the AT50 of IgG and N-Ab responses were longer following the third dose than after the second dose. Other studies [18] have also demonstrated a similar pattern where waning of both IgG and N-Ab was slower after the third compared to the second dose (IgG 1.32% vs. 2.26% per day and N-Ab 1.32% vs. 3.34% per day). Given that total S-Ab exhibited a shorter AT50 than IgG post-booster, both antibody levels may need to be assessed to observe the waning antibody levels post-vaccination, and IgG may be the preferred antibody to follow-up for long-term antibody response.

As all antibodies decline with time after vaccination, an important question is what would be a suitable antibody level which may be considered protective and when that point is reached. This is difficult to establish since other factors besides quantifiable antibody titers (such as memory B cell responses) can contribute to protective immunity [19]. Nevertheless, some have tried to estimate this protective level. One report found a positive correlation between protection against infection and increasing IgG levels, with 67% protection at 94 BAU/mL using the OmniPATH 384 Combi SARS-CoV-2 IgG ELISA assay [20]. The estimated mean duration to the point of 67% protection was 161–227 days. At 270 days, almost all subjects fell below the level of 67% protection. In another study, an overall IgG level of 154 BAU/mL (MSD SARS-Coronavirus IgG assay) was considered protective, derived from a reverse cumulative distribution model, averaging from all thresholds across six different vaccination regimens [21]. Some go further to state that a vaccine efficacy of 80% against symptomatic COVID-19 was achieved with an even higher threshold of 264 BAU/mL for IgG S-Ab (MSD), and a 247 normalized neutralization titer for the live-virus neutralization assay (pseudovirus neutralizing antibody assay, validated at Monogram Biosciences), with the risk of symptomatic COVID-19 decreasing with increasing levels of IgG S-Abs [22]. Although more work is needed before the two assays can be compared [23], the findings show that both IgG S-Ab and neutralization titers are associated with increased vaccine efficacy. Furthermore, both the Abbott IgG and Roche total S-Ab assays used in our study have been shown to correlate well with virus neutralization assays (about 3.5–8.5 weeks after the first positive PCR in COVID-19 patients, Abbott r = 0.90 and Roche r = 0.83 with the virus neutralization assay normalized neutralization titers) [24]. In our population, median IgG antibody levels fell to 246 BAU/mL by 120–150 days after the second vaccination, 141 BAU/mL by 180–210 days after vaccination, and to 102 BAU/mL by 240 days after vaccination. Thus, a booster vaccination might be required by roughly 60–90 days post-second dose to maintain a possible “protective” level of antibodies, and at 90 days post-second dose the IgG S-Ab titers fell below 600 BAU/mL. Indeed, one study [25] found that a suitably robust micro-neutralization test titer (≥1:80) was only achieved at Abbott IgG levels of 1814–2000 BAU/mL in vaccinated individuals. However, in our study, 180–210 days post-booster, the IgG S-Ab titers had fallen to a median of 401 BAU/mL. In our study, the AT50 of all antibodies seemed to lie within the region of 60–100 days post-booster. Thus, some have proposed that after 16–20 weeks (112–140 days), a fourth booster dose may be required to maintain antibody levels [26]. 

Although the third vaccine dose generated significantly higher antibody peaks than the second dose, the rise in total S-Ab was more pronounced after the third vaccination, as evidenced by the higher total:IgG S-Ab ratios. In one study, the booster dose generated a significantly higher IgG S-Ab than the second dose (180 vs. 147 EIA units), with a ratio of 1.22 [27]. Our two IgG peaks had a ratio of 1.29. Our population demonstrated a third:second dose total S-Ab ratio of 8.81. In some other studies, the ratio can be even as high as 26.6 (peak median S-Ab levels were 284 vs. 7554 AU/mL), although their study was not able to clearly exclude asymptomatic/pauci-symptomatic COVID-19 cases [28]. In another study that assessed the Roche total S-Ab after second and third vaccinations [29], the third:second dose S-Ab ratio was also very high at 10.4 (21,657 vs. 2082 U/mL) 2 weeks after the third and second doses of mRNA vaccine. While the total S-Ab does exhibit a greater-fold rise post-booster than IgG compared to responses to the second inoculation, the exact physiological cause of this phenomenon would require further study. Furthermore, we also found that the higher post-booster total S-Ab may not be due to the development of post-booster IgM since peak post-booster IgM S-Ab was significantly lower than the peak titers after the second dose. The decrease in IgM post-booster is a new finding in our study, and further studies would be required to see if the IgM levels post-booster rise earlier than 20–30 days post-booster. Other studies also show that IgM responses are less robust in vaccinated individuals compared to patients with COVID-19 infection [30]. Further studies [31] have also shown that in seropositive individuals (with previous COVID-19), immunization did not elicit significantly raised IgM responses (S1, S2, and RBD). Furthermore, in seronegative individuals, only S1 IgM antibodies had a significant increase after a second dose [31]. These findings lend some support to the IgM antibody trends in our study, and they may indicate that there may be some other immunological mechanism in the initial induction of IgM that is difficult to boost further by repeated exposure. However, this does indicate that compared to IgG/total S-Ab/N-Ab, IgM would be less preferred for monitoring antibody responses post-booster.

Although we were unable to compare the effect of the decrease in antibody titers over time after booster vaccinations with waning vaccine effectiveness, several reports have studied the pattern over time. One study showed that up to 3 months post-booster, the vaccine effectiveness for homologous BNT162b2 schedules was up to 95.3% [32]. In another study [33], the booster vaccine had an effectiveness of 67.2% at 2–4 weeks against the Omicron variant but declined to 45.7% by 10 weeks (70 days). It has also been reported that post-booster vaccine effectiveness decreased from 53.4 to 16.5% in just a matter of three months [34]. All these studies seem to suggest a correlation between waning vaccine effectiveness and decreasing antibody titers. Further studies are needed to determine the precise relationship between the two. 

In summary, our study reports the following new findings:

1. Peak antibody levels after booster vaccination were significantly higher than the second vaccination in all antibody levels except IgM; total S-Ab and N-Ab had the most pronounced increase;
2. Total S-Ab, IgG S-Ab, and N-Ab all decline over a period of 210 days after booster vaccination. Antibody titers 210 days after the booster dose were still significantly higher than pre-booster titers;
3. The half-lives of IgG and N-Ab were longer post-booster than after the second vaccination.

A limitation of our study is that we have comparatively fewer post-booster vaccinees than after dose two. However, we have demonstrated that the antibody kinetics are in agreement with those of other studies with larger cohorts. We are unable to correlate our findings with vaccine effectiveness during the various “waves” from 2021 to 2022. However, one local study [13] showed that during the Omicron wave (December 2021 onwards), mRNA booster effectiveness 15–60 days post-booster was as high as 31.7%. Currently, we do not have data on the antibody levels after 210 days post-booster vaccination. There is also variability between the antibody levels reported by different total S-Ab/IgG/N-Ab assays [35], and IgG/total S-Ab/N-Ab results may not be generalizable to other analytical platforms. However, the conversion of total S-Ab and IgG to WHO international standardized units should go some way to improving the comparability of results.

## 5. Conclusions

In conclusion, all total S-Ab/IgG/N-Ab antibodies showed a robust increase in levels after a third booster vaccination, with improved peak antibody titers compared to the second dose. A booster vaccination resulted in IgG and N-Ab antibodies that displayed a greater half-life than the second vaccination. However, all antibody titers displayed declined over time, which may indicate the need for a fourth booster dose, especially in vulnerable populations at the appropriate time.

## Figures and Tables

**Table 1:** Mean, Median and range of total spike, IgG spike, neutralizing and IgM spike antibody responses to 3 doses of Pfizer vaccine.

Time Point	Roche Total Spike Antibodies				Abbott IgG Spike Antibodies				Snibe Neutralizing Antibodies				Abbott IgM Spike Antibodies			
	n	Mean (BAU/mL)	Median (BAU/mL)	Range (BAU/mL)	n	Mean (BAU/mL)	Median (BAU/mL)	Range (BAU/mL)	n	Mean (ug/mL)	Median (ug/mL)	Range (ug/mL)	n	Mean (COI)	Median (COI)	Range (COI)
Baseline	73	0.40	0.39	0.39–3.60	72	0.64	0.62	0.04–21.1	53	0.016	0.018	0–0.11	73	0.043	0.030	0.01–0.77
Dose 1 D10	78	3.70	3.29	0.39–677	78	19.9	17.8	0.78–850	74	0.062	0.068	0–1.71	76	0.33	0.38	0.02–7.55
Dose 2 D20	72	2048	2219	146–11,194	72	2101	2270	217–7764	69	3.61	3.52	0.51–30	70	2.02	2.11	0.13–13.6
Dose 2 D40	51	1438	1695	274–3633	50	1443	1547	185–5963	51	2.77	2.84	0.39–30	51	1.21	1.32	0.09–15.8
Dose 2 D60	51	1315	1454	226–4353	51	887	941	111–2549	50	2.34	2.35	0.74–6.93	50	0.72	0.86	0.07–2.96
Dose 2 D90	40	1132	1259	233–4318	40	496	542	110–1482	40	1.76	1.95	0.45–7.14	38	0.36	0.39	0.06–1.99
Dose 2 D120–150	32	1025	1275	219–2852	29	234	246	74.7–733	31	1.24	1.64	0.37–2.83	28	0.19	0.15	0.02–1.92
Dose 2 D180–210	16	637	695	114–2936	14	158	141	27.0–2452	16	0.81	0.93	0.19–1.92	14	0.12	0.10	0.02–1.57
Dose 2 D240–PreD3	71	521	502	39.0–2804	71	103	102	13.2–680	70	0.62	0.61	0.09–2.48	63	0.12	0.10	0.02–3.48
Dose 3 D20–30	42	19,150	19,551	3803–357,552	41	2634	2932	128–6084	40	20.9	26.4	1.41–185	25	0.29	0.23	0.06–4.33
Dose 3 D60	9	12,968	14,992	1730–45,830	9	2201	2659	267–6296	9	15.9	20.6	2.34–35.1	2	0.18	0.18	0.05–0.64
Dose 3 D90	63	7739	7532	1157–31,000	59	1154	1276	254–4520	59	11.3	12.5	2.45–40.7	–	–	–	–
Dose 3 D120–150	18	4970	6349	1009–16,636	18	694	873	98.8–2208	17	7.81	9.73	0.98–21.9	–	–	–	–
D3D180–210	50	3498	3353	460–51,539	43	490	401	27.0–2452	47	5.81	5.41	0.88–33.2	–	–	–	–

## Supplementary Materials

The following supporting information can be downloaded at: https://www.mdpi.com/article/10.3390/vaccines10101703/s1, Supplementary Table S1: STARD checklist.

## Abbreviations

S-Ab	Spike antibodies
N-Ab	Neutralizing antibodies
AT50	Half life
